# MLOmics: Cancer Multi-Omics Database for Machine Learning

**DOI:** 10.1038/s41597-025-05235-x

**Published:** 2025-05-30

**Authors:** Ziwei Yang, Rikuto Kotoge, Xihao Piao, Zheng Chen, Lingwei Zhu, Peng Gao, Yasuko Matsubara, Yasushi Sakurai, Jimeng Sun

**Affiliations:** 1 Bioinformatics Center, Institute for Chemical Research, Kyoto University, Kyoto, Japan; 2 SANKEN, Osaka University, Osaka, Japan; 3 IRCN, The University of Tokyo, Tokyo, Japan; 4 Institute for Quantitative Biosciences, The University of Tokyo, Tokyo, Japan; 5 Department of Computer Science, University of Illinois Urbana-Champaign, Champaign, USA; 6 Carle Illinois College of Medicine, University of Illinois Urbana-Champaign, Champaign, USA

**Keywords:** Machine learning, Cancer genomics

## Abstract

Framing the investigation of diverse cancers as a machine learning problem has recently shown significant potential in multi-omics analysis and cancer research. Empowering these successful machine learning models are the high-quality training datasets with sufficient data volume and adequate preprocessing. However, while there exist several public data portals, including The Cancer Genome Atlas (TCGA) multi-omics initiative or open-bases such as the LinkedOmics, these databases are not off-the-shelf for existing machine learning models. In this paper, we introduce MLOmics, an open cancer multi-omics database aiming at serving better the development and evaluation of bioinformatics and machine learning models. MLOmics contains 8,314 patient samples covering all 32 cancer types with four omics types, stratified features, and extensive baselines. Complementary support for downstream analysis and bio-knowledge linking are also included to support interdisciplinary analysis.

## Background & Summary

Multi-omics analysis has shown great potential to accelerate cancer research. A promising trend consists of framing the investigation of diverse cancers as a machine learning problem, where complex molecular interactions and dysregulations associated with specific tumor cohorts are revealed through integration of multi-omics data into machine learning models. Several impressive achievements have been demonstrated in molecular subtyping^1–3^, disease-gene association prediction^4–6^, and drug discovery^7^.

Empowering successful machine learning models are the high-quality training datasets with sufficient data volume and adequate preprocessing. While there exist several public data portals including The Cancer Genome Atlas (TCGA) multi-omics initiative^8^ or open-bases such as the LinkedOmics^9^, these databases are not off-the-shelf for existing machine learning models. To make these data model-ready, a series of laborious, task-specific processing steps such as metadata review, sample linking, and data cleaning are mandatory. The domain knowledge required, as well as a deep understanding of diverse medical data types and proficiency in bioinformatics tools have become an obstacle for researchers outside of such backgrounds. The gap between the growing body of powerful machine learning models and the absence of well-prepared public data has become a major bottleneck. Currently, some existing methods validate their proposed machine learning models using inconsistent experimental protocols, with variations in datasets, data processing techniques and evaluation strategies^10^. These studies could benefit from a fair assessment of extensive baselines on a uniform footing with unified datasets and to offer more reliable recommendations on models.

**Fig. 1:**
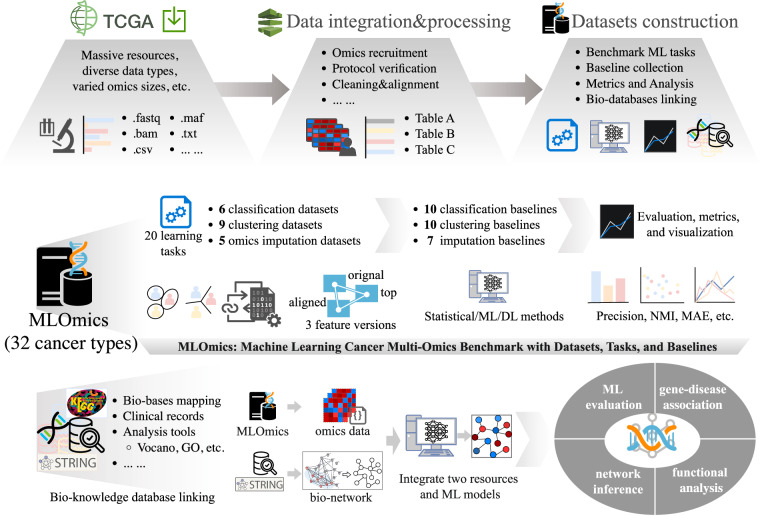
Schematic workflow of creating the MLOmics. The process starts with collecting patient samples covering 32 cancer types from the TCGA project. All resources in diverse data types and sizes are uniformly integrated and processed to contain data of four omics types. Datasets for benchmark ML tasks were constructed based on the processed data. MLOmics also selected baselines, metrics, and resources to support downstream biological analysis. **Overview of the MLOmics**. MLOmics provides an interface for developing and evaluating machine learning models based on cancer multi-omics data. MLOmics provides datasets in three feature scales for 20 classification, clustering, and omics imputation learning tasks. MLOmics also provides statistical, ML, and DL baselines for each task, which are evaluated by fair metrics. **Bio-knowledge database linking with MLOmics**. MLOmics provides resources to link with other bio-knowledge databases, enabling the integration of outer resources for applications such as ML evaluation, gene-disease association exploration, network inference, and functional analysis.

To meet the growing demand of the community, we introduce MLOmics, an open cancer multi-omics database aiming at serving better the development and evaluation of bioinformatics and machine learning models. MLOmics collected 8,314 patient samples covering all 32 cancer types from the TCGA project. All samples were uniformly processed to contain four omics types: mRNA expression, microRNA expression, DNA methylation, and copy number variations, followed by categorization, protocol verification, feature profiling, transformation, and annotation. For each dataset, we provide three feature versions: Original, Aligned, and Top, to support feasible analysis. For example, the Top version contains the most significant features selected via the ANOVA test^11^ across all samples to filter out potentially noisy genes. The MLOmics datasets were carefully examined with 6 ~ 10 highly cited baseline methods. These baselines were rigorously reproduced and evaluated with various metrics to ensure fair comparison. Complementary resources are included to support basic downstream biological analysis, such as clustering visualization, survival analysis, and Volcano plots. Last but not least, we provide support to interdisciplinary analysis via our locally deployed bio-base resources. Interdisciplinary researchers can retrieve and integrate bio-knowledge of cancer omics studies through resources such as the STRING^12^ and KEGG^13^. For instance, exploration of bio-network inference^14^ and simulated gene knockouts^15^ is supported. In summary, MLOmics is an open, unified database approachable to non-experts for developing/evaluating machine learning models; conducting interdisciplinary analysis and supporting cancer research and broader biological studies. We showcase the above usages in the Usage Notes section. A detailed overview of the MLOmics database and its characteristics is provided in Fig. 1.

## Methods

### Data Collection and Preprocessing

The data were sourced from TCGA via the Genomic Data Commons (GDC) Data Portal^16^. The original resources in TCGA are organized by cancer type, and the omics data for individual patients are scattered across multiple repositories. Therefore, retrieving and collecting omics data require linking samples with metadata and applying different preprocessing protocols. MLOmics employs a unified pipeline that integrates data preprocessing, quality control, and multi-omics assembly for each patient, followed by alignment with their respective cancer types. Specifically, we perform different steps for each omics type as follows:

**Transcriptomics (mRNA and miRNA) data:** 1. *Identifying transcriptomics*. We trace the downloaded data using the “experimental_strategy” field in the metadata, marked as “mRNA-Seq” or “miRNA-Seq”, then we verify that “data_category” is labeled as “Transcriptome Profiling.” 2. *Determining experimental platform*. We identify the experimental platform from the metadata, such as “platform: Illumina” or “workflow_type: BCGSC miRNA Profiling.” 3. *Converting gene-level estimates*. For data generated by platforms like Illumina Hi-Seq, use the edgeR package^17^ to convert scaled gene-level RSEM estimates into FPKM values. 4. *Non-Human miRNA filtering*. For “miRNA-Seq” data from platforms like Illumina GA and Agilent arrays, we identify and remove non-human miRNA expressions using species annotations from databases such as miRBase^18^. 5. *Noise eliminating*. We remove features with zero expression in more than 10% of samples or those with undefined value (N/A). 6. *Transformation*. Finally, we apply logarithmic transformations to obtain the log-converted mRNA and miRNA data.

**Genomic (CNV) data:** 1. *Identifying CNV Alterations*. We examine how gene copy-number alterations are recorded in the metadata, using key descriptions such as “Calls made after normal contamination correction and CNV removal using thresholds.” 2. *Filter Somatic Mutations*. We capture only somatic variants by retaining entries marked as “somatic” and filtering out germline mutations. 3. *Identify Recurrent Alterations*. We use the GAIA package^19^ to identify recurrent genomic alterations in the cancer genome, based on raw data representing all aberrant regions from copy-number variation segmentation. 4. *Annotate Genomic Regions*. We annotate the recurrent aberrant genomic regions using the BiomaRt package^20^.

**Epigenomic (Methy) data:** 1. *Identify Methylation Regions*. We examine how methylation is defined in metadata to map methylation regions to genes, using key descriptions like “Average methylation (beta-values) of promoters defined as 500bp upstream & 50 downstream of Transcription Start Site (TSS)” or “With coverage >= 20 in 70% of the tumor samples” 2. *Normalize Methylation Data*. We perform median-centering normalization to adjust for systematic biases and technical variations across samples, using the R package limma^21^. 3. *Select Promoters with Minimum Methylation*. For genes with multiple promoters, we select the promoter with the lowest methylation levels in normal tissues.

After processing the omics sources, the data are annotated with unified gene IDs to resolve variations in naming conventions caused by the difference in sequencing methods or reference standards^22^. Then, the omics data are aligned across multiple sources based on their corresponding sample IDs. Finally, the data files are organized by cancer type for further dataset construction.

### Datasets Construction

MLOmics reorganizes the collected and processed data resources into different feature versions tailored to various machine learning tasks. For each task, MLOmics provides several baselines, evaluation metrics, and the ability to link with biological databases such as STRING and KEGG for further biological analysis of different machine learning models.

#### Feature Processing

Machine learning models require tabular data with a the same number of features across samples. In addition to the Original feature scale that contains a full set of genes (variations included) directly extracted from the collected omics files, MLOmics provides two other well-processed feature scales: Aligned and Top. The former scale filters out non-overlapping genes and selects the genes shared across different cancer types; and the latter identifies the most significant features. Specifically, the following steps are performed for each scale:

**Aligned:** 1. we resolve the mismatches in gene naming formats such as ensuring compatibility between cancers that use different reference genomes. 2. we identify the intersection of feature lists across datasets to ensure all selected features are present in different cancers. 3. we conduct z-score feature normalization.

**Top:** 1. we perform multi-class ANOVA^11^ to identify genes with significant variance across multiple cancer types. 2. we perform multiple testing using the Benjamini-Hochberg (BH)^23^ correction to control the false discovery rate (FDR)^24^. 3. the features are ranked by the adjusted *p*-values *p* < 0.05 or by the user-specified scales). 4. we conduct z-score feature normalization which reduces the presence of non-significant genes across cancers and this could be beneficial for biomarker studies.

#### 20 Task-ready Datasets with Baselines and Metrics

We provide 20 off-the-shelf datasets ready for machine learning models ranging from pan-cancer/cancer subtype classification, subtype clustering to omics data imputation. We also include well recognized baselines that leveraged classical statistical approaches and machine/deep learning methods as well as metrics for standard evaluation.

**Pan-cancer and golden-standard cancer subtype classification**. Pan-cancer classification aims to identify each patient’s specific cancer type. Moreover, a cancer typically comprises multiple subtypes that differ in their biochemical profiles. Some subtypes have been well-studied in prior research and widely accepted as the golden standard. We re-label patient samples to support subtyping evaluation. These two classification tasks potentially improve cancer early diagnostic accuracy and treatment outcomes.

*Datasets:* MLOmics provides six labeled datasets: one pan-cancer dataset and five gold-standard subtype datasets (GS-COAD, GS-BRCA, GS-GBM, GS-LGG, and GS-OV).

*Baselines:* we opt for the following classical classification methods as baselines: XGBoost^25^, Support Vector Machines (SVM)^26^, Random Forest (RF)^27^, and Logistic Regression (LR)^28^. Additionally, we include six popular, open-sourced deep learning methods: Subtype-GAN^29^, DCAP^30^, XOmiVAE^31^, CustOmics^32^, and DeepCC^33^.

*Metrics:* For classification evaluation, we opt for precision (Pre), recall (Re), and F1-score (F1). Since clustering is the primary focus of the subtyping task, due to the limited sample size (typically <100), we provide normalized mutual information (NMI) and adjusted rand index (ARI) to evaluate the agreement between the clustering results obtained by different methods and the true labels.

**Cancer Subtype Clustering**. Cancer subtyping remains an open question under fierce debate for most cancers, especially rare types. Numerous studies propose various clustering methods to identify distinct groups by identifying different clusters to support downstream evaluation and discovery of new subtypes.

*Datasets:* MLOmics provides nine unlabeled rare cancer datasets (ACC, KIRP, KIRC, LIHC, LUAD, LUSC, PRAD, THCA, and THYM) for this learning task.

*Baselines:* In addition to the aforementioned Subtype-GAN, DCAP, MAUI, XOmiVAE, we also include six clustering methods: Similarity Network Fusion (SNF)^34^, Neighborhood-based Multi-Omics clustering (NEMO)^35^, Cancer Integration via Multi-kernel Learning (CIMLR)^36^, iClusterBayes^37^, moCluster^38^, and MCluster-VAEs^39^.

*Metrics:* To evaluate the goodness of clustering we opt for the classic Silhouette coefficient (SIL)^40^ and log-rank test *p*-value on survival time (LPS)^41^.

**Omics Data Imputation**. In addition to classification and clustering, we also provide a data imputation task focusing on imputing multi-omics cancer data. The collected omics data typically have missing values due to experimental limitations, technical errors, or inherent variability. The imputation process is crucial for ensuring the integrity and usability of TCGA omics data^42^.

*Datasets:* MLOmics provides five omics datasets with missing values (Imp-BRCA, Imp-COAD, Imp-GBM, Imp-LGG, and Imp-OV). Given a full dataset as a matrix $D\in {{\mathbb{R}}}^{n\times m}$, we follow prior works^42,43^ to generate a mask matrix *M* ∈ {0, 1}^*n*×*m*^ uniformly at random with the probability of missing *P*(*M*_*ij*_ = 0) = *r*_miss_, and the probability of retaining *P*(*M*_*ij*_ = 1) = 1 − *r*_miss_. The final data matrix is obtained by multiplying element-wise the data matrix *D* with the mask *M*. The missing level *r*_miss_ is selected from [0.3, 0.5, 0.7].

*Baselines:* We opt for seven well-recognized methods for imputing missing values, including: Mean Imputation that imputes the missing entry with mean values of entries around it (Mean); K-Nearest Neighbors (KNN) that imputes the missing value with the weighted Euclidean K nearest neighbors; Multivariate Imputation by Chained Equations (MICE) that performs multiple regression to model each missing value conditioned on non-missing values^44^; Iterative SVD (iSVD) that imputes by iterative low-rank SVD decomposition^45^; Spectral Regularization Algorithm (Spectral) that also employs SVD but with a soft threshold and the nuclear norm regularization^46^; Generative Adversarial Imputation Nets (GAIN) that proposes to distinguish between fake and true missing patterns by the generator-discriminator architecture^43^; Graph Neural Network for Tabular Data (GRAPE) that utilizes the graph networks to impute based on learned information from columns and rows of the data matrix^42^.

*Metrics:* We use the Mean Squared Error (MSE) between the unmasked entries (*M*_*ij*_ = 1) as the training loss to let the model predict the actual value. During the test, the masked missing values are used for evaluating the model performance (*M*_*ij*_ = 0).

MLOmics will be continuously updated with baselines and evaluations on the defined learning tasks. A detailed description of the baselines and metrics is provided in the Supplementary Material.

## Data Records

The MLOmics main datasets^47^ are available on Figshare (https://figshare.com/articles/dataset/MLOmics_Cancer_Multi-Omics_Database_for_Machine_Learning/28729127) and Hugging Face (https://huggingface.co/datasets/AIBIC/MLOmics). The MLOmic main datasets are now accessible under the Creative Commons 4.0 Attribution (CC-BY-4.0), which supports its use for educational and research purposes. The main datasets presented include all cancer multi-omics datasets corresponding to the various tasks described above.

### MLOmics Overview

The MLOmics repository is structured into three sections: (1) Main Datasets, (2) Baseline and Metrics, and (3) Downstream Analysis Tools and Resources Linking.

The core section is the **(1) Main Datasets:** the repository hosts a comprehensive collection of tasks-ready cancer multi-omics datasets, stored primarily as .csv (comma-separated values) files.

Along with the other two sections: (2) Baseline and Metrics: the repository provides source codes of baseline models and evaluation metrics for different tasks, typically implemented in Python or R code; (3) Downstream Analysis Tools and Resources Linking: the repository encompasses additional tools and resources that complement the main datasets and downstream omics analysis needs, implemented as .csv, .py or R files.

In the following parts of this section, we focus on presenting the properties of the **Main Datasets**, detailing its organizational structure, principal components, and resources.

### Main Datasets Format

Main datasets of MLOmics use .csv files to manage and store all omics datasets, widely favored in biomedical research, including multi-omics studies. Despite its simplicity, .csv remains efficient even with large datasets encountered in genomic and proteomic studies. Both Python and R provide built-in functions and libraries to read, write, and analyze .csv files efficiently.

Specifically, MLOmics provides its main datasets in .csv files as plain-text files, where commas separate data values. These files maintain a structured and intuitive format, with rows representing individual data records and columns corresponding to different attributes or variables. The key characteristics of the main dataset files are:

- **Encoding:** UTF-8, ensuring broad compatibility across different systems and software.
- **Delimiter:**, (comma), used to separate values in each row.
- **Line Ending:** LF (\n) for Unix/Linux or CRLF (\r\n) for Windows, ensuring proper formatting across operating systems.
- **Header Row:** The first row contains column names, typically representing sample identifiers (e.g., Sample1, Sample2, Sample3, etc.).
- **Data Rows:** Each subsequent row corresponds to a distinct feature, such as a gene, with attributes like expression values recorded across multiple patient samples.
- **Values:** Numeric values (float), representing continuous measurements of omics attributes.

For example, consider a simplified .csv file containing mRNA data for a set of gene features (rows) across several patient samples (columns):

**Table:** 

**Feature**	**Sample1**	**Sample2**	**Sample3**	**Sample4**
GeneA	0.23	0.18	0.35	0.21
GeneB	0.56	0.49	0.52	0.58
GeneC	0.19	0.22	0.15	0.17
GeneD	0.08	0.10	0.09	0.12

In this example, each row corresponds to a specific gene (GeneA, GeneB, GeneC, GeneD). Each column represents a different sample (Sample1, Sample2, Sample3, Sample4). The numeric values (variables) in the cells denote the expression levels of each gene in each sample.

### Main Datasets Structure

**Fig. 2:**
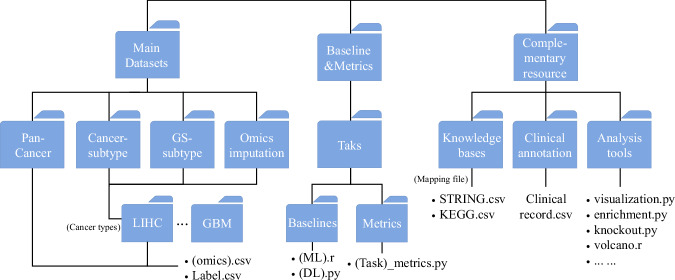
Schematic of MLOmics resources structure. The MLOmics framework consists of three major components. The Main Datasets file includes all cancer multi-omics datasets for various tasks. The Baseline and Metrics file provides the source code for baseline models and performance metrics. The Downstream Analysis Tools and Resources Linking file offers sources for further analysis and links to additional biological resources.

As shown in Fig. 2, the main datasets repository is organized into three layers:

**(1) The first layer** contains three files corresponding to three different tasks: Classification_datasets, Clustering_datasets, and Imputation_datasets.

**(2) The second layer** includes files for specific tasks, such as GS-BRCA, ACC, and Imp-BRCA.

**(3) The third layer** contains three files corresponding to different feature scales, i.e., Original, Aligned, and Top.

The omics data from different omics sources are stored in the following files: Cancer_miRNA_Feature.csv, Cancer_mRNA_Feature.csv, Cancer_CNV_Feature.csv, and Cancer_Methy_Feature.csv. Here, Cancer represents the cancer type, and Feature indicates the feature scale type.

The ground truth labels are provided in the file Cancer_label_num.csv, where Cancer represents the cancer type. The patient survival records are stored in the file Cancer_survival.csv.

## Technical Validation

**Fig. 3:**
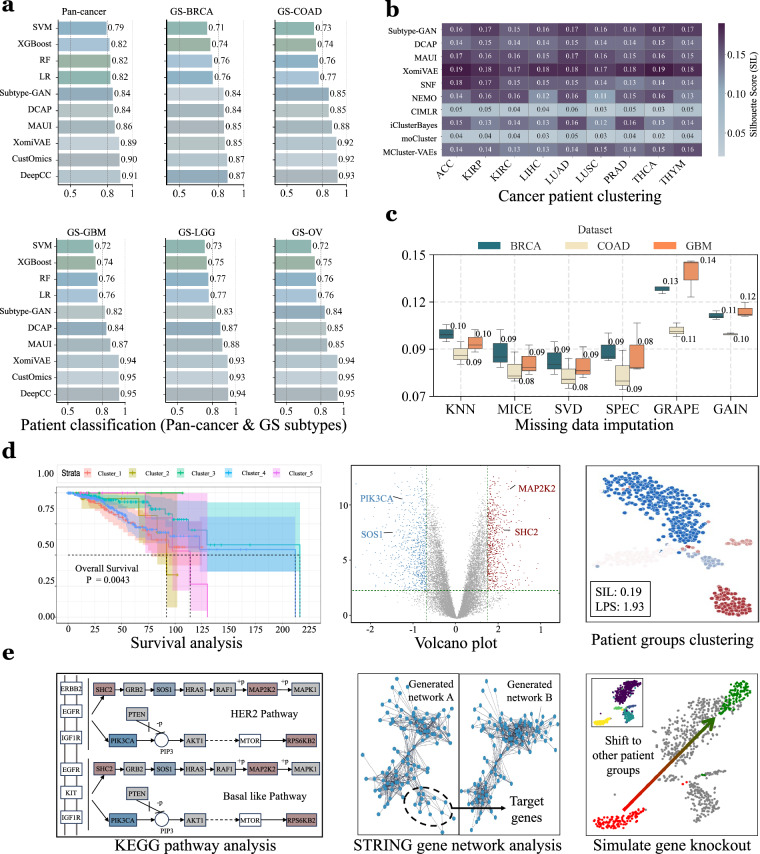
Experimental results and downstream analyses of machine learning baselines applied to MLOmics datasets. **(a)** PREC bar plots for each baseline method across all datasets. Overall, machine learning-based methods (Subtype-GAN, DCAP, MAUI, XOmiVAE, CustOmics, and DeepCC) outperformed traditional statistical methods (SVM, XGBoost, RF, and LR). **(b)** SIL heatmaps for each baseline method across all datasets. Methods employing deep generative neural network architectures (Subtype-GAN, DCAP, MAUI, XOmiVAE, and MCluster-VAEs) generally outperformed other methods (SNF, NEMO, CIMLR, iClusterBayes, and moCluster). **(c)** Box plots for each baseline method across three imputation datasets. Matrix decomposition methods (SVD, Spectral) outperformed deep learning-based methods (GAIN, GNN). **(d,e)** Schematic illustrations of downstream analysis results based on the clustering outcomes of XOmiVAE applied to specific cancer patient clustering datasets. In the survival analysis plot, survival curves in different colors correspond to distinct clustering groups. In the volcano plot and KEGG pathway analysis, red and blue indicate downregulated and upregulated genes between patient groups, respectively. In the patient group clustering plot, different colors represent samples belonging to different clusters. In the simulated gene knockout analysis, red and green dots indicate sample clustering before and after expression knockout, respectively.

The Methods section detailed the data collection, preprocessing and curation of the MLOmics datasets. To validate the collected datasets, we set up a series of classification, clustering and imputation experiments each with a wide array of models ranging from conventional statistical models to deep neural networks. The experimental results and evaluations are summarized in Fig. 3.

## Usage Notes

**Fig. 4:**
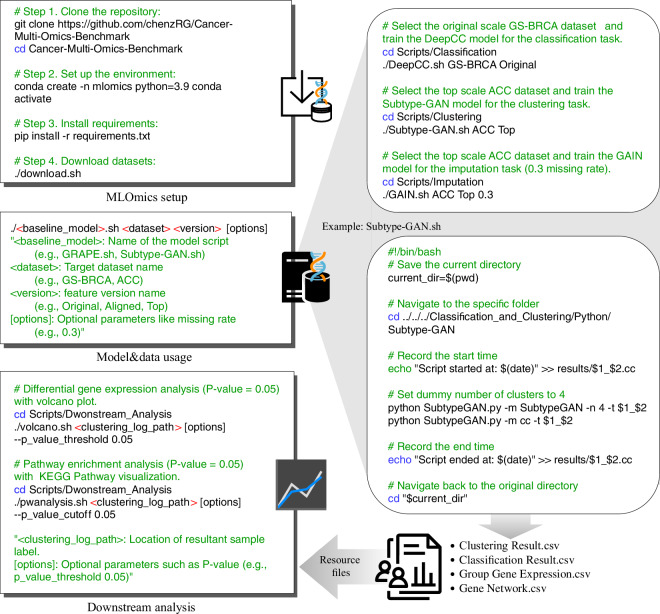
The code usage note of MLOmics. The MLOmics can be used following the illustrated code usage instructions, including MLOmics setup, code usage for custom model-task-dataset specifications, and code for downstream analysis workflows.

We provide comprehensive guidelines for utilizing the MLOmics repository, as illustrated in Fig. 4. All codes and files are ready for direct loading and analysis using standard Python data packages such as NumPy and Pandas.

As a demonstration of how to use MLOmics for machine learning applications, we provide 20 classification, clustering, and imputation tasks with fair evaluation protocols for pan-cancer analysis, cancer subtypes, and omics imputation. Access to these resources is provided in the Code Availability section. A rising trend in multi-omics analysis is to integrate multi-omics data (non-network data) with biological networks to better understand complex functions on the gene or protein level. MLOmics provides offline linking resources for well-established databases such as STRING^12^ and KEGG^13^. We hope it can lower the barriers to entry for machine learning researchers interested in developing methods for cancer multi-omics data analysis, thereby encouraging rapid progress in the field.

## Supplementary information

Supplementary Material

## Data Availability

All codes and resources of MLOmics are publicly available under the CC-BY-4.0. The code for preprocessing and preparing the main MLOmics databases, as well as the benchmarking algorithms used in MLOmics, is available at the following repository: (https://github.com/chenzRG/Cancer-Multi-Omics-Benchmark).
