# Supplementary material for: MLOmics: Cancer Multi-Omics Database for Machine Learning
Source: Sci Data. 2025 May 30;12:913. doi: 10.1038/s41597-025-05235-x (PMC12125382; doi:10.1038/s41597-025-05235-x)
Supplement: Supplementary file 1 — Supplementary Material [file 41597_2025_5235_MOESM1_ESM.pdf]

Supplementary Material

Contents

A Key Information about MLOmics **2**
A.1 MLOmics Structure 2
A.2 Recruited Cancer 3
A.3 Recruited Omics 4

B MLOmics Preprocessing Pipelines **5**
B.1 For Transcriptomics (mRNA and miRNA) Data 5
B.2 For Genomic (CNV) Data 5
B.3 For Epigenomic (Methy) Data 5

C MLOmics Feature Scale Processing **6**
C.1 Original Features 6
C.2 Aligned Features 6
C.3 Top Features 6

D MLOmics Tasks **8**
D.1 Pan-cancer Classification 8
D.2 Cancer Subtype Clustering 8
D.3 Golden-standard Subtype Classification 8
D.4 Omics Data Imputation 8

E MLOmics Evaluation Metrics **9**
E.1 Precision (Pre) 9
E.2 Recall (Re) 9
E.3 F1-Score (F1) 9
E.4 Normalized Mutual Information (NMI) 9
E.5 Adjusted Rand Index (ARI) 9
E.6 Silhouette Coefficient (SIL) 9
E.7 P-value of the log-rank Test on Survival Time (LPS) 10
E.8 Mean Absolute Error (MAE) 10
E.9 Root Mean Squared Error (RMSE) 10

F Downstream Analysis and Biological Resources Linking **11**
F.1 Differential Gene Expression Analysis 11
F.2 Survival Analysis 11
F.3 KEGG Pathway Analysis 11
F.4 STRING Network Mapping 11
F.5 Simulate Gene Knockout 12

G Data Source Ethics and Policies **13**
G.1 Human Subjects Protection and Data Access Policies 13
G.2 Data Use Certification Agreement 13
G.3 Suggested Informed Consent Language for Prospective Collections 13
G.4 Sharing Data from Large-scale Biological Research Projects 13
G.5 Considerations for Open Release of Genomic Data from Human Cancer Cell Lines 13

H Limitations & Broader Impact **14**

References **15**

A Key Information about MLOmics

A.1 MLOmics Structure

Here, we present the organizational structure of the MLOmics, detailing its main components and resources. The MLOmics repository is structured into three primary sections: **Main Datasets**, **Baseline and Metrics**, and **Downstream Analysis Tools and Resources Linking**.

```
MLOmics
├── Main Datasets
│   ├── Pan-Cancer Dataset [Classification_datasets]
│   │   └── Pan-Cancer
│   │       └── Aligned
│   │           └── mRNA / miRNA / CNV / Methy / Label.csv
│   │
│   ├── Cancer Subtype Datasets [Clustering_datasets]
│   │   ├── ACC
│   │   ├── KIRP
│   │   ├── KIRC
│   │   ├── LIHC
│   │   ├── LUAD
│   │   ├── LUSC
│   │   ├── PRAD
│   │   ├── THCA
│   │   └── THYM
│   │   (ABOVE) └── Original & Aligned & Top
│   │               └── mRNA / miRNA / CNV / Methy.csv
│   │
│   ├── Golden-Standard Cancer Subtype Datasets [Classification_datasets]
│   │   ├── GS-COAD
│   │   ├── GS-BRCA
│   │   ├── GS-GBM
│   │   ├── GS-LGG
│   │   └── GS-OV
│   │   (ABOVE) └── Original & Aligned & Top
│   │               └── mRNA / miRNA / CNV / Methy / Label.csv
│   │
│   ├── Omics Data Imputation Datasets [Imputation_datasets]
│   │   ├── Imp-COAD
│   │   ├── Imp-BRCA
│   │   ├── Imp-GBM
│   │   ├── Imp-LGG
│   │   └── Imp-OV
│   │   (ABOVE) └── Top
│   │               └── mRNA / miRNA / CNV / Methy.csv
│   │
├── Baseline Models and Metrics
│   ├── Classification Tasks
│   │   ├── Baselines.py
│   │   └── Metrics.py
│   │
│   ├── Clustering Tasks
│   │   ├── Baselines.py/r
│   │   └── Metrics.py
│   │
│   └── Omics Data Imputation Tasks
│       ├── Baselines.py
│       └── Metrics.py
│
└── Downstream Analysis Tools and Resources Linking
```

```
    ├── Knowledge_bases
    │   └── STRING_mapping / KEGG_mapping.csv
    │
    ├── Clinical Annotation
    │   └── Clinical_Rec.csv
    │
    └── Analysis Tools
        └── Analysis_Tools.py/r
```

A.2 Recruited Cancer

The MLOmics database contains multi-omics data for 32 types of cancer. The full names and abbreviations as shown in the following table:

No.	Full Name	Abbreviation
1	Acute Myeloid Leukemia	LAML
2	Adrenocortical Cancer	ACC
3	Bladder Urothelial Carcinoma	BLCA
4	Brain Lower Grade Glioma	LGG
5	Breast Invasive Carcinoma	BRCA
6	Cervical & Endocervical Cancer	CESC
7	Cholangiocarcinoma	CHOL
8	Colon Adenocarcinoma	COAD
9	Diffuse Large B-cell Lymphoma	DLBC
10	Esophageal Carcinoma	ESCA
11	Head & Neck Squamous Cell Carcinoma	HNSC
12	Kidney Chromophobe	KICH
13	Kidney Clear Cell Carcinoma	KIRC
14	Kidney Papillary Cell Carcinoma	KIRP
15	Liver Hepatocellular Carcinoma	LIHC
16	Lung Adenocarcinoma	LUAD
17	Lung Squamous Cell Carcinoma	LUSC
18	Mesothelioma	MESO
19	Ovarian Serous Cystadenocarcinoma	OV
20	Pancreatic Adenocarcinoma	PAAD
21	Pheochromocytoma & Paraganglioma	PCPG
22	Prostate Adenocarcinoma	PRAD
23	Rectum Adenocarcinoma	READ
24	Sarcoma	SARC
25	Skin Cutaneous Melanoma	SKCM
26	Stomach Adenocarcinoma	STAD
27	Testicular Germ Cell Tumor	TGCT
28	Thymoma	THYM
29	Thyroid Carcinoma	THCA
30	Uterine Carcinosarcoma	UCS
31	Uterine Corpus Endometrioid Carcinoma	UCEC
32	Uveal Melanoma	UVM

Table 1. Cancer Types and Abbreviations in MLOmics

A.3 Recruited Omics

MLOmics recruited four types of omics data:

- *mRNA* (mRNA expression) measures the levels of messenger RNA transcribed from genes, reflecting the active transcription of genetic information;
- *miRNA* (miRNA expression) quantifies the levels of microRNAs and small non-coding RNA molecules. It is crucial for post-transcriptional regulation in gene expression;
- *Methy* (DNA methylation) measures the addition of methyl groups to DNA, typically at cytosine bases. It influences gene expression by altering the DNA accessibility to transcriptional machinery.
- *CNV* (copy number variations) represents variations in the number of copies of particular DNA segments. This omics affects gene dosage and contributes to cancer susceptibility.

B MLOmics Preprocessing Pipelines

Here are the details for processing different omics:

B.1 For Transcriptomics (mRNA and miRNA) Data

1. **STEP 1: Identify Transcriptomics Data** Trace the data by "experimental_strategy" in the metadata, marked as "mRNA-Seq" or "miRNA-Seq". Check if "data_category" is marked as "Transcriptome Profiling".

2. **STEP 2: Determine Experimental Platform** Identify the experimental platform from metadata, such as "platform: Illumina" or "workflow_type: BCGSC miRNA Profiling".

3. **STEP 3: Convert Gene-Level Estimates** For data from the Hi-Seq platform like Illumina, use the R package edgeR[1] to convert the scaled estimates in the original gene-level RSEM to FPKM.

4. **STEP 4: Filter Non-Human miRNA** For "miRNA-Seq" data from Illumina GA and Agilent array platforms, identify and remove non-human miRNA expression features using species annotation from databases like miRBase[2].

5. **STEP 5: Eliminate Noise** Identify and eliminate features with zero expression levels in more than 10% of samples or missing values (designated as N/A).

6. **STEP 6: Apply Logarithmic Transformation** Apply a logarithmic transformation to get the log-converted mRNA and miRNA data.

B.2 For Genomic (CNV) Data

1. **STEP 1: Identify CNV Alterations in Metadata** Examine how alterations in gene copy-number are recorded in metadata using key descriptions like "Calls made after normal contamination correction and CNV removal using thresholds."

2. **STEP 2: Filter Somatic Mutations** Use keyword filtering to capture only somatic mutations, excluding germline mutations by retaining only those marked as 'somatic."

3. **STEP 3: Identify Recurrent Alterations** Use the R package GAIA[3] to identify recurrent alterations in the cancer genome from raw data that denote all aberrant regions resulting from copy number variation segmentation.

4. **STEP 4: Annotate Genomic Regions** Use the R package BiomaRt[4] to annotate the aberrant recurrent genomic regions.

5. **STEP 5: Save Annotated CNV Data** Save the annotated results to get CNV data of significantly amplified or deleted genes.

B.3 For Epigenomic (Methy) Data

1. **STEP 1: Identify Methylation Regions in Metadata** Examine how methylation is defined in metadata to map methylation regions to genes, using key descriptions like "Average methylation (beta-values) of promoters defined as 500bp upstream & 50 downstream of Transcription Start Site (TSS)" or "With coverage >= 20 in 70% of the tumor samples and 70% of the normal samples."

2. **STEP 2: Normalize Methylation Data** Implement a median-centering normalization to account for systematic biases and technical variations across samples using the R package limma[5].

3. **STEP 3: Select Promoters with Minimum Methylation** For genes with multiple promoters, select the promoter with minimum methylation in the normal tissues.

4. **STEP 4: Save Mapped Methylation Data** Save the mapped value data where each entry corresponds to a specific gene or genomic region, along with corresponding methylation measurements.

C MLOmics Feature Scale Processing

Cancer multi-omics analysis often suffers from data issues, such as unbalanced sample sizes and feature dimensions.

To address this, after the typical omics data preprocessing, MLOmics provides three versions of feature scales (*Original*, *Top*, and *Aligned*) to support different machine learning tasks. Table 2 summarizes the different feature scales' details.

C.1 Original Features

The original features are genes directly extracted from each preprocessed omics dataset. It represents the complete, full-size set of patients' gene features without any task-specific filtering. This version supports users in customizing their datasets based on their specific requirements, such as re-filtering to target gene sets or omics-specific transformations.

Key technical operations in generating original features include:

1. Retaining all genes after normalization (e.g., log transformation or z-score normalization).
2. Imputing missing values using methods like K-nearest neighbors (KNN) or median imputation.
3. Filtering out low-quality samples (e.g., samples of low-variances or high missing genes).

C.2 Aligned Features

Aligned features are the intersection of genes common to all datasets for a learning task, representing the shared features in different cancer types. This reduces the original feature size, ensures consistency in multi-omics features, and primarily provides support across cancer-type studies.

Key technical operations for generating aligned features include:

1. Resolving unmatches in gene naming formats (e.g., ensuring compatibility between cancers using different references genome).
2. Identifying the intersection of feature lists across datasets to ensure all selected features are present in different cancers.
3. Normalization features(e.g., log transformation or z-score normalization).

C.3 Top Features

Top features are selected based on ANOVA[6] statistical testing, ranked by p-values to identify the most significant features across cancers. The default top feature scale settings for mRNA, miRNA, methylation, and CNV data are 5000, 200, 5000, and 5000, respectively, with significance determined by $p < 0.05$. This approach greatly reduces noisy genes across cancers and achieves smaller feature-dimension cancer datasets, making them suitable for feature-dimension-sensitive machine-learning models.

Additional key technical operations for generating top features include:

1. Performing multi-class ANOVA to identify genes with significant variance across multiple cancer types.
2. Adjusting for multiple testing using the Benjamini-Hochberg correction to control the false discovery rate (FDR).
3. Ranking features by adjusted p-values and selecting the top k features per omics type, as defined by the default or user-specified scales.
4. Normalization features(e.g., log transformation or z-score normalization).

The detailed feature size of different MLOmics datasets is below:

Table 2. MLOmics provides multiple feature scales for nine unlabeled cancer subtype datasets and five labeled, golden-standard subtype datasets.

Dataset	Feature Scale	Omics Feature Size			
		mRNA	miRNA	Methy	CNV
ACC	Orignal	18204	368	19045	19525
	Aligned	10452	254	10347	10154
	Top	5000	200	5000	5000
KIRP	Orignal	17254	375	19023	19532
	Aligned	10452	254	10347	10154
	Top	5000	200	5000	5000
KIRC	Orignal	18464	352	19045	19523
	Aligned	10452	254	10347	10154
	Top	5000	200	5000	5000
LIHC	Orignal	17945	435	19053	19523
	Aligned	10452	254	10347	10154
	Top	5000	200	5000	5000
LUAD	Orignal	18303	435	19034	19532
	Aligned	10452	254	10347	10154
	Top	5000	200	5000	5000
LUSC	Orignal	18577	745	19025	19543
	Aligned	10452	254	10347	10154
	Top	5000	200	5000	5000
PRAD	Orignal	17954	467	19034	19534
	Aligned	10452	254	10347	10154
	Top	5000	200	5000	5000
THCA	Orignal	17480	345	19024	19532
	Aligned	10452	254	10347	10154
	Top	5000	200	5000	5000
THYM	Orignal	18341	535	19034	19532
	Aligned	10452	254	10347	10154
	Top	5000	200	5000	5000
GS-COAD	Orignal	18234	462	19023	19545
	Aligned	11343	286	11189	11203
	Top	5000	200	5000	5000
GS-BRCA	Orignal	18233	345	19053	19533
	Aligned	11343	286	11189	11203
	Top	5000	200	5000	5000
GS-GBM	Orignal	17545	335	19034	19545
	Aligned	11343	286	11189	11203
	Top	5000	200	5000	5000
GS-LGG	Orignal	18345	345	19023	19534
	Aligned	11343	286	11189	11203
	Top	5000	200	5000	5000
GS-OV	Orignal	1735	244	19034	19534
	Aligned	11343	286	11189	11203
	Top	5000	200	5000	5000

Table 3. Summary of all tasks in the MLOmics under each task category.

Entry	Task Categories	Task Names
1	Pan-cancer Classification	Pan-cancer
2	Golden-standard Subtype Classification	GS-BRCA, GS-COAD, GS-GBM, GS-LGG, GS-OV
3	Cancer Subtype Clustering	ACC, KIRP, KIRC, LIHC, LUAD, LUSC, PRAD, THCA, THYM
4	Omics Data Imputation	Imp-BRCA, Imp-COAD, Imp-GBM, Imp-LGG, Imp-OV

D MLOmics Tasks

The MLOmics database contains multi-omics data curated for a range of task categories, as summarized in Table 3. Definitions for each task category are provided below.

D.1 Pan-cancer Classification

Let $X^O = \{x_1, x_2, ..., x_m\}$ represent the multi-omics dataset, where each x_i is a vector of features in O-th omics for the i-th sample. Let Y denote the set of possible cancer types. The goal of cancer classification using multi-omics data is to predict the true label y_i for each sample x_i in X, where y_i belongs to the set of possible cancer types Y. Cancer classification can be formulated as a supervised learning problem, where the objective is to learn a mapping function $f : X \rightarrow Y$ that accurately predicts the true labels for unseen samples based on their omics features.

D.2 Cancer Subtype Clustering

Cancer subtyping means categorizing patients into subgroups that exhibit differences in various aspects based on their multi-omics data. However, for most cancer types, especially rare cancers, the cancer subtyping tasks are still open questions under discussion. Thus cancer subtyping tasks are typically clustering tasks without ground true labels. Let $X^O = \{x_1, x_2, ..., x_m\}$ represent the multi-omics dataset, where each x_i is a vector of features in O-th omics for the i-th sample. Let k denote the set of possible cancer subtypes. The goal of cancer subtyping using multi-omics data is to assign each sample x_i in X into k clusters $C = \{C_1, C_2, ..., C_k\}$, such that each cluster C_i represents a distinct cancer subtype based on the information from multiple omics data sources.

D.3 Golden-standard Subtype Classification

The cancer research community has thoroughly analyzed the subtypes of some of the most common cancer types in a previous study. Therefore, we consider these subtypes to be the true labels. The definition of golden-standard subtype identification is similar to the above Pan-cancer identification tasks. Golden-standard subtype identification task aims to assign each sample x in the sample set X to a cancer subtype y in the set of all subtypes Y.

D.4 Omics Data Imputation

Let X denote the original omics data with m samples and n features, represented as a matrix where X_{ij} represents the value of the i-th sample for the j-th feature. Let M denote the binary mask matrix of the same dimensions as X, where $M_{ij} = 1$ if the value of X_{ij} is observed (not missing), and $M_{ij} = 0$ if it is missing. The goal of the imputation task is to estimate the missing values in X, denoted as $\hat{X}$, using the observed values and potentially additional information. Imputation can be formulated as $\hat{X} = f(X, M)$, where f is the imputation function that takes as input the original omics data X and the mask matrix M, and outputs the imputed matrix $\hat{X}$.

E MLOmics Evaluation Metrics

E.1 Precision (Pre)

Precision measures the accuracy of the positive predictions made by a classification or clustering model. It is defined as the ratio of true positive (TP) predictions to the total number of positive predictions made by the model:

$$Pre = \frac{TP}{TP + FP}$$

where TP is the number of true positive predictions (instances correctly classified as positive), and FP is the number of false positive predictions (instances incorrectly classified as positive).

E.2 Recall (Re)

Recall, also known as sensitivity, measures the ability of a classification or clustering model to identify all relevant instances (i.e., TP) correctly. It is defined as the ratio of TP predictions to the total number of actual positive instances:

$$Re = \frac{TP}{TP + FN}$$

where TP is the number of true positive predictions (instances correctly classified as positive), and FN is the number of false negative predictions (instances incorrectly classified as negative).

E.3 F1-Score (F1)

The F1-score is the harmonic mean of precision and recall, and it provides a balanced measure of a model's accuracy by considering both false positives and false negatives. It is particularly useful when the dataset is imbalanced. The F1-score is calculated as:

$$F1 = 2 \cdot \frac{Pre \cdot Re}{Pre + Re}$$

where Pre is precision, and Re is recall.

E.4 Normalized Mutual Information (NMI)

Normalized mutual information measures the similarity between two clusterings of the same dataset. It measures the mutual dependence between the clustering result and the ground truth labels, normalized by the average entropy of the two clusterings. Let C be the clustering result and G be the ground truth labels. Then, NMI is calculated as:

$$NMI(C,G) = \frac{I(C,G)}{\sqrt{H(C) \cdot H(G)}}$$

where $I(C,G)$ is the mutual information between C and G, $H(C)$ and $H(G)$ are the entropies of C and G, respectively.

E.5 Adjusted Rand Index (ARI)

Adjusted rand index measures the similarity between two clusterings of the same dataset. It measures the agreement between the pairs of samples assigned to the same or different clusters in the two compared clusterings, adjusted for chance. ARI is calculated as:

$$ARI(C,G) = \frac{a+b}{\binom{n}{2}} - \frac{a \cdot (a-1) + b \cdot (b-1)}{\binom{n}{2}}$$

where a is the number of pairs of samples that are in the same cluster in both C and G, b is the number of pairs of samples that are in different clusters in both C and G, n is the total number of samples, and $\binom{n}{2}$ is the number of all possible pairs of samples.

E.6 Silhouette Coefficient (SIL)

The silhouette coefficient measures the similarity between a sample and its classified subtype compared to the samples in the other subtypes to determine how appropriately samples in a dataset have been clustered. For a sample i, let $a(i)$ be the average distance from sample i to other samples in the same cluster, and let $b(i)$ be the smallest average distance from sample i to samples in a different cluster, minimized over clusters. The silhouette coefficient $SIL(i)$ for a sample i is then defined as:

$$SIL(i) = \frac{b(i) - a(i)}{\max\{a(i), b(i)\}}$$

The silhouette coefficient ranges from -1 to 1, where a high value indicates that the sample is well-matched to its own cluster and poorly matched to neighboring clusters.

E.7 P-value of the log-rank Test on Survival Time (LPS)

The log-rank test on survival time is a hypothesis test used to compare the survival distributions of two or more groups. The test statistic X^2 is calculated from the observed and expected number of events in each group over time. The p-value is then calculated from the test statistic under the null hypothesis that there is no difference in survival distributions between the groups. The LPS gives the log-transformed p-values of the log-rank test. It is calculated as below using the chi-square distribution with $k-1$ degrees of freedom:

$$LPS = P(X^2 \geq X^2_{observed})$$

where k is the number of groups being compared, $X^2_{observed}$ is the observed test statistic calculated from the data.

E.8 Mean Absolute Error (MAE)

Mean absolute error measures the average absolute difference between the imputed values and the true values as below:

$$MAE = \frac{1}{n}\sum_{i=1}^{n}|\hat{Y}_i - Y_i|$$

where n is the number of imputed values, $\hat{Y}_i$ is the imputed value for observation i and Y_i is the true value for observation i.

E.9 Root Mean Squared Error (RMSE)

Root mean squared error measures the square root of the average squared difference between the imputed values and the true values as below:

$$RMSE = \sqrt{\frac{1}{n}\sum_{i=1}^{n}(\hat{Y}_i - Y_i)^2}$$

where n is the number of imputed values, $\hat{Y}_i$ is the imputed value for observation i and Y_i is the true value for observation i.

F Downstream Analysis and Biological Resources Linking

F.1 Differential Gene Expression Analysis

Differential gene expression analysis has been a cornerstone of transcriptomic studies. In this analysis, we compare gene expression levels between different experimental conditions or sample groups to identify genes that are significantly upregulated or downregulated. Statistical tests such as t-tests or non-parametric tests are commonly used for this purpose. For example, gene expression profiles between cancer patients and healthy controls can be compared to identify genes that are dysregulated in cancer. Genes with significant differences in expression levels may be further investigated as potential biomarkers or therapeutic targets. For example, researchers performed differential gene expression analysis on RNA-seq data from Alzheimer's disease patients and healthy controls. This analysis identified a panel of differentially expressed genes implicated in neuroinflammation and synaptic dysfunction, showing molecular pathways associated with Alzheimer's disease progression.

We calculated the log2 fold change in gene abundance between pairwise groups and determined the significance of expression changes using Student's t-test. P-values were adjusted using the Benjamini-Hochberg procedure to correct the false discovery rate. We considered a gene to be significant if it had an adjusted p-value less than 0.05 and a log2 fold change greater than or equal to 1.2. Based on their fold changes, the resulting DEGs were categorized into up-regulated and down-regulated sets and can be utilized for subsequent analysis phases.

Among the identified DEGs, several genes have been extensively reported as being associated with cancer progression. Notable examples include BRCA1, WNT4, and NOTCH2. BRCA1 is well-known for its involvement in hereditary breast cancer and plays essential roles in cell cycle regulation, DNA damage response, and transcriptional control[7]. Dysregulation of the WNT4 gene, which encodes a protein belonging to the Wnt signaling pathway, has been linked to tumor growth, invasion, and metastasis[8]. Similarly, the NOTCH2 gene, a member of the Notch receptor family, is critical in cell fate determination, development, and tissue homeostasis and has been implicated in tumor initiation, progression, and therapy resistance[9].

F.2 Survival Analysis

Survival analysis is a vital statistical method used to examine and interpret the time until the occurrence of an event, such as death, disease progression, or relapse, in clinical studies. It provides insights into factors that influence the survival probability of patients and helps in understanding the impact of clinical, demographic, and molecular variables on patient outcomes. Common survival analysis techniques include the Kaplan-Meier estimator for survival curves and the Cox proportional hazards model for assessing the relationship between survival and multiple covariates.

In MLOmics, survival analysis is conducted using time-to-event data, such as patient survival time and event status (alive/dead or disease-free/relapsed). In our approach, we use log-rank tests to compare survival curves between different groups and assess the significance of survival differences. Multivariate Cox regression is employed to evaluate the combined effect of multiple factors on survival. Adjustments for confounding variables and interactions are made, and results are presented with hazard ratios and corresponding confidence intervals. Survival analysis results are visualized using Kaplan-Meier survival curves.

F.3 KEGG Pathway Analysis

Pathway analysis is a critical step in interpreting the biological significance of DEGs. By mapping DEGs to known biological pathways, researchers can gain insights into the underlying mechanisms and potential functional impacts of gene expression changes. In MLOmics, pathway analysis is performed using established databases such as KEGG. These databases provide curated information on metabolic pathways, signaling cascades, and gene ontologies. DEGs are input into pathway analysis tools to conduct the analysis, which then identifies overrepresented pathways among the upregulated and downregulated gene sets.

For instance, pathway enrichment analysis might reveal that upregulated DEGs in cancer samples are significantly associated with pathways involved in cell cycle regulation and apoptosis, while downregulated DEGs are linked to immune response pathways. Such findings can help to identify potential therapeutic targets and elucidate the molecular basis of disease.

In our approach, we utilize Fisher's exact test or hypergeometric test to evaluate the significance of pathway enrichment. Adjustments for multiple testing are performed using the Benjamini-Hochberg procedure, with pathways considered significant at an adjusted p-value threshold of less than 0.05. The pathway analysis results are visualized using enrichment plots and pathway diagrams, which highlight key genes and interactions within the enriched pathways.

F.4 STRING Network Mapping

The STRING database[10] aggregates PPIs from experimental data, computational predictions, and curated datasets, offering a standardized framework for network analysis. STRING network mapping is used to identify and analyze

protein-protein interactions (PPIs) among differentially expressed genes. This approach facilitates the identification of hub nodes and key interaction pathways in different patients and disease groups. For example, patient clusters often correspond to functional modules or biological pathways, leading to different gene networks.

In omics analyses, gene identifiers often differ across databases and platforms, which can pose challenges in integrating data for downstream analyses. Omics datasets may use Ensembl IDs, Entrez IDs, or gene symbols, while the STRING database requires its own set of identifiers to query PPIs. This step is essential for maintaining data consistency and enabling precise network analysis: without proper mapping, some genes might be excluded from the analysis due to identifier mismatches, leading to incomplete or biased results.

In MLOmics, a mapping file resolves these discrepancies by linking MLOmics gene identifiers from omics data to their corresponding STRING identifiers. This mapping file is a CSV format file that contains two columns. The first column is gene identifiers used in the MLOmics dataset. The second column provides the matching STRING identifiers required for querying the STRING database. This structure ensures a straightforward lookup for identifier conversion. Moreover, the CSV format makes inspecting, updating, and adapting this mapping file for other workflows or databases easy.

Once identifiers are mapped, DEGs can be input into the STRING database to construct interaction networks and further network visualization, typically performed with node attributes (e.g., gene expression values or statistical significance) and edge attributes (e.g., interaction confidence) encoded in the visualization.

F.5 Simulate Gene Knockout

The simulation begins by ranking all genes based on node degree disparities calculated from the connectivity matrices of the sub-networks. Node degree is quantified as the number of direct connections each gene has to other genes within the network, serving as a measure of its centrality and influence across different cancer subtypes. To derive the connectivity matrices, we analyze the interactions between genes, where each gene is represented as a node and each interaction as an edge. The degree of each node is then computed to identify highly interconnected genes.

After ranking, we categorize the genes into two sets: a *high-ranking gene set*, which includes genes exhibiting the largest degree disparities (above a defined threshold based on node degree variance), and a *low-ranking gene set*, composed of genes with minimal degree differences (below the same threshold). Using node degree variance as a threshold ensures our classification is statistically grounded. This method isolates genes that play critical roles in the network dynamics.

Next, we individually simulate the knockout of genes within the high-ranking and low-ranking gene sets. This process involves transforming their expression values to a baseline non-expression level, which is defined as either zero or a predefined low expression value (such as the mean expression level of the lowest 10% of genes). This transformation mimics the functional loss of these genes. For each gene target in the selected sets, we systematically replace its expression value in the patient samples with the baseline non-expression level.

G Data Source Ethics and Policies

The ultimate goal of data source ethics and policies was to develop research policies maximizing public benefit from the data that were by these ethical and legal guidelines, ensuring: (1) Protection of human participants in the project, including their privacy; (2) Secure and compliant access to TCGA data; (3) Timely data release to the research community; (4) Initial scientific publication by the data producers; (5) These policies have influenced the field of cancer genomics and will continue to serve as a guide for future genomic research projects.

G.1 Human Subjects Protection and Data Access Policies

NCI and NHGRI developed a set of policies to protect the privacy of participants donating specimens to TCGA. TCGA's informed consent policy, data access policy, and information about compliance with the HIPAA Privacy Rule are included.

G.2 Data Use Certification Agreement

Researchers must agree to A set of policies before gaining access to TCGA data. This agreement ensures that researchers pursuing a research question requiring controlled-access data comply with TCGA policies, such as maintaining participants' privacy, securely accessing the data, and following TCGA publication guidelines.

G.3 Suggested Informed Consent Language for Prospective Collections

An example informed consent document that TCGA suggested Tissue Source Sites use when collecting specimens from prospective project participants. This document helps ensure that patients considering donating tissue specimens to human genomics research programs such as TCGA recognize the risks and benefits of participation and understand the nature of their inclusion in the project.

G.4 Sharing Data from Large-scale Biological Research Projects

Principles for sharing and publishing genomic data to maximize public benefit developed at a meeting in Fort Lauderdale sponsored by the Wellcome Trust. These "Fort Lauderdale Principles" informed the original TCGA publication guidelines, which balance making genomic data immediately available for research use with protecting the original owner's initial publication rights.

G.5 Considerations for Open Release of Genomic Data from Human Cancer Cell Lines

An explanation of the factors considered in the decision by NCI and NHGRI to release genomic data and information from the Cancer Cell Line Encyclopedia as open-access data.

H Limitations & Broader Impact

This research proposes a benchmark for cancer multi-omics data analysis. However, we collected all data from TCGA sources and did not conduct wet experiments to introduce new data further. Consequently, the data is limited and influenced by the specific cohorts and methodologies used in TCGA, which may not fully represent the diversity of cancer types or the broader patient population. Cancer omics data also raises ethical issues, particularly in cancer risk prediction and the development of anti-cancer drugs, which could have potentially harmful or controversial functions. The use of omics data for predictive purposes can lead to concerns about privacy, discrimination, and the psychological impact on individuals who are identified as high-risk. Additionally, designing drugs based on omics data can lead to unintended side effects and ecological impacts if not carefully regulated.

Nevertheless, we believe that omics data has great potential to benefit society. It can lead to more personalized and effective treatments, early cancer detection, and a better understanding of cancer biology. Negative impacts can be mitigated through stringent industry regulations, ethical guidelines, and legislation to ensure responsible use and data protection. The proposed benchmark helps the community develop new cancer omics data analysis algorithms and evaluate the performance of existing models. By providing a standardized framework, we aim to facilitate advancements in cancer research and improve the reproducibility and comparability of different computational approaches.

References

1. Robinson, M. D., McCarthy, D. J. & Smyth, G. K. edger: a bioconductor package for differential expression analysis of digital gene expression data. *bioinformatics* **26**, 139–140 (2010).
2. Kozomara, A., Birgaoanu, M. & Griffiths-Jones, S. mirbase: from microrna sequences to function. *Nucleic acids research* **47**, D155–D162 (2019).
3. al. SMe. *gaia: GAIA: An R package for genomic analysis of significant chromosomal aberrations* (2021). R package version 2.39.0.
4. Durinck, S. *et al.* Biomart and bioconductor: a powerful link between biological databases and microarray data analysis. *Bioinformatics* **21**, 3439–3440 (2005).
5. Ritchie, M. E. *et al.* limma powers differential expression analyses for rna-sequencing and microarray studies. *Nucleic acids research* **43**, e47–e47 (2015).
6. St, L., Wold, S. *et al.* Analysis of variance (anova). *Chemom. intelligent laboratory systems* **6**, 259–272 (1989).
7. Miki, Y. *et al.* A strong candidate for the breast and ovarian cancer susceptibility gene brca1. *Science* **266**, 66–71, 10.1126/science.7545954 (1994).
8. Peradziryi, H. *et al.* Wnt signaling in development and disease. *Prog. Mol. Biol. Transl. Sci.* **153**, 87–153, 10.1016/B978-0-12-385928-0.00003-3 (2011).
9. Gridley, T. Notch signaling and inherited disease syndromes. *Hum. Mol. Genet.* **12**, R9–R13, 10.1093/hmg/ddg076 (2003).
10. Szklarczyk, D. *et al.* The string database in 2023: protein–protein association networks and functional enrichment analyses for any sequenced genome of interest. *Nucleic acids research* **51**, D638–D646 (2023).
